# Centers for Disease Control (CDC) Wound Classification is Prognostic of 30-Day Readmission Following Surgery

**DOI:** 10.1007/s00268-023-07093-3

**Published:** 2023-07-05

**Authors:** Victoria Yin, J. Perren Cobb, Sean C. Wightman, Scott M. Atay, Takashi Harano, Anthony W. Kim

**Affiliations:** 1 Keck School of Medicine, University of Southern California, 1975 Zonal Avenue, Los Angeles, CA 90033 USA; 2 Departments of Surgery & Anesthesiology, Critical Care Institute, Keck School of Medicine, University of Southern California, 1520 San Pablo Street, Suite 4300, Los Angeles, CA 90033 USA; 3 Division of Thoracic Surgery, Department of Surgery, Keck School of Medicine, University of Southern California, 1510 San Pablo Street, Suite 514, Los Angeles, CA 90033 USA

## Abstract

**Background:**

The goal of this study was to investigate factors associated with 30-day readmission in a multivariate model, including the CDC wound classes “clean,” “clean/contaminated,” “contaminated,” and “dirty/infected.”

**Methods:**

The 2017–2020 American College of Surgeons-National Surgical Quality Improvement Program (ACS-NSQIP) database was queried for all patients undergoing total hip replacement, coronary artery bypass grafting, Ivor Lewis esophagectomy, pancreaticoduodenectomy, distal pancreatectomy, pneumonectomy, and colectomies. ACS-defined wound classes were concordant with CDC definitions. Multivariate linear mixed regression was used to determine risk factors for readmission while adjusting for type of surgery as a random intercept.

**Results:**

477,964 cases were identified, with 38,734 (8.1%) patients having experienced readmission within 30 days of surgery. There were 181,243 (37.9%) cases classified as wound class “clean”, 215,729 (45.1%) cases classified as “clean/contaminated”, 40,684 cases (8.5%) classified as “contaminated”, and 40,308 (8.4%) cases classified as “dirty/infected”. In the multivariate generalized mixed linear model adjusting for type of surgery, sex, body mass index, race, American Society of Anesthesiologists class, presence of comorbidity, length of stay, urgency of surgery, and discharge destination, “clean/contaminated” (*p* < .001), “contaminated” (*p* < .001), and “dirty/infected” (*p* < .001) wound classes (when compared to “clean”) were significantly associated with 30-day readmission. Organ/space surgical site infection and sepsis were among the most common reasons for readmission in all wound classes.

**Conclusions:**

Wound classification was strongly prognostic for readmission in multivariable models, suggesting that it may serve as a marker of readmissions. Surgical procedures that are “non-clean” are at significantly greater risk for 30-day readmission. Readmissions may be due to infectious complications; optimizing antibiotic use or source control to prevent readmission are areas of future study.

**Supplementary Information:**

The online version contains supplementary material available at 10.1007/s00268-023-07093-3.

## Introduction

Hospital readmissions after surgeries can be costly and unnecessary. To improve hospital care and penalize higher than predicted readmission rates, the Center for Medicare and Medicaid Services (CMS) created the Hospital Readmission Reduction Program [1, 2]. Despite studies showing mixed results regarding readmission as a reliable indicator for quality of care, readmission remains an important marker of hospital quality as it is used by CMS [2, 3]. Indicators for elevated risk of hospital readmission among surgical patients are of interest to physicians and hospitals to ensure quality patient care and avoid penalties associated with readmission.

**Fig. 1:**
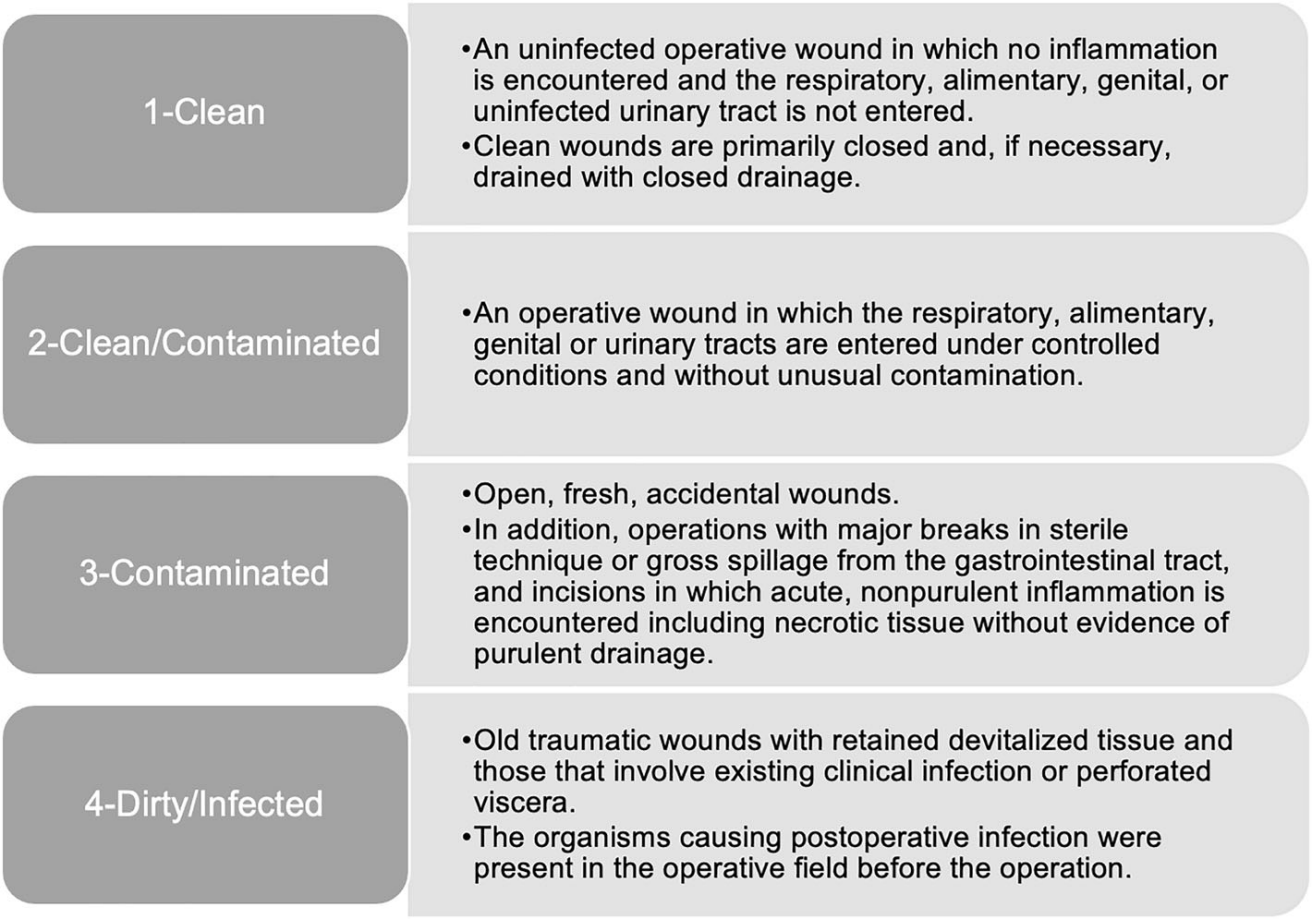
Wound class definitions from the American college of surgeons [9]

Wound class, a variable recorded in all surgeries and determined during the operation, is often used to estimate a patient’s risk for postoperative surgical site infection (SSI) [4–8]. Definitions provided by the American College of Surgeons (ACS) for the National Surgical Quality Improvement Program (NSQIP) database for each wound class: clean, clean/contaminated, contaminated, and dirty/infected, are presented in Fig. 1 [9]. These definitions are concordant with Centers of Disease Control (CDC) definitions [10]. Previous studies have shown that wound classifications higher or “dirtier” than “clean” are at increased risk for postoperative complications and mortality [7, 8, 11]. However, these studies did not examine whether wound classification was an important factor for readmission following surgeries of diverse wound classes. It is unknown whether wound classification is significantly associated with risk of readmission among a larger, broader population of surgical patients.

The objective of this study was to evaluate CDC wound classification as a prognostic factor for thirty-day readmission following surgery. We hypothesized that “clean/contaminated,” “contaminated,” and “dirty/infected” wound classes, when compared to “clean,” would have significantly greater risk for thirty-day readmission in a multivariate analysis of all total hip replacement, coronary artery bypass grafting (CABG), Ivor Lewis esophagectomy, pancreaticoduodenectomy, distal pancreatectomy, pneumonectomy, or colectomy in a nationwide surgical database.

## Materials and methods

### Data source

The ACS NSQIP, a large, national database of surgeries and up to 30-day postoperative outcomes, was utilized for this study [12]. All patients undergoing total hip replacement, CABG, Ivor Lewis esophagectomy, pancreaticoduodenectomy, distal pancreatectomy, pneumonectomy, and colectomies in the years 2017 through 2020 were isolated. Total hip replacement and coronary artery bypass grafting were included because they are currently penalized under the CMS Hospital Readmission Reduction Program [1]. The remaining surgeries were selected to provide diversity in wound class within the sample while having similar typical hospital length of stay. All Current Procedural Terminology (CPT) codes used to identify the surgeries are listed in Supplementary Table 1. Wound class definitions from the American College of Surgeons (concordant with CDC definitions) are provided in Fig. 1 [9]. Patients who did not have data regarding their wound class or hospital readmission status or had miscoded wound class (by definitions given in Fig. 1) were excluded from this study (Supplementary Fig. 1). This study was approved by the University of Southern California Institutional Review Board.

### Variables

The ACS NSQIP captures readmission status up to 30 days after the principal operation; this was used as the primary outcome of the study. Data on age, gender, body mass index (BMI), preoperative comorbidities, principal operation, wound classification, American Society of Anesthesiologists (ASA) class, 30-day readmission status, discharge destination, post-operative complications, hospital length of stay, urgency of surgery, suspected reason for readmission, and 30-day mortality were collected. Comorbidities indexed by ACS NSQIP include diabetes, current smoking status, ventilator dependency, history of chronic obstructive pulmonary disease, ascites, history of congestive heart failure, hypertension, acute renal failure, steroid use for chronic condition, bleeding disorders, and preoperative sepsis; patients with any of these diagnoses were considered to have comorbidity. Postoperative infectious complication was defined as presence of wound infection (which includes organ/space, deep, and superficial SSI), pneumonia, urinary tract infection, or sepsis, as these are outcomes specifically captured by ACS NSQIP for each patient.

### Statistical analysis

Univariate analysis was conducted using Student’s t-test or Wilcoxon Rank Sum test for continuous variables and Chi-square test for categorical variables. Statistical significance was defined as p-value less than 0.05. The statistical programs “glm” and “glmer” with R version 4.2.1 were utilized to conduct the data analysis [13]. Significant fixed effect covariates for readmission were identified using multivariate logistic regression with backward stepwise selection. Then, multivariate generalized linear mixed regression was performed to model readmission with adjustment for type of surgery as a random effect and wound class, sex, BMI (centered on mean and standardized), race, ASA class, discharge destination, presence of comorbidities, urgency of surgery and length of stay (centered on mean and standardized) as fixed effects. Modeling type of surgery as a “random intercept” accounts for readmission risk specific to certain procedures, while all other independent variables were assumed to have a consistent effect on readmission risk across all types of surgery as “fixed effects”. Absence of collinearity among independent variables was confirmed by measuring variance inflation factors. Normality of the random intercept, type of surgery, was confirmed using the Shapiro–Wilk test. Multivariate logistic regression models for postoperative complications controlled for wound class, BMI, sex, race, ASA class, urgency of surgery, type of surgery, and presence of comorbidities.

## Results

### Sample demographics and characteristics

There were 477,964 cases meeting inclusion criteria, and of those, 38,734 (8.1%) patients experienced readmission within 30 days of surgery. There were 181,243 cases (37.9%) classified as “clean” wound class, 215,729 (45.1%) cases classified as “clean/contaminated,” 40,684 cases (8.5%) classified as “contaminated,” and 40,308 (8.4%) cases classified as “dirty/infected” (Table 1). In our sample, the readmission rate by wound class was 3.9% (6,986 of 181,243) for “clean” surgeries, 10.3% (22,145 of 215,729) for “clean/contaminated” surgeries, 12.0% (4,871 of 40,684) for “contaminated” surgeries, and 11.7% (4,732 of 40,308) for “dirty/infected” surgeries. In the overall cohort, readmitted patients had a statistically significant longer hospital stay than patients who did not experience readmission (p < 0.001). Sex, race, age, BMI, wound class, type of surgery, urgency of surgery, ASA class, presence of comorbidity, and postoperative infectious complications (wound infection, pneumonia, urinary tract infection, or sepsis) were significantly associated with readmission status. 30-day mortality was not significantly associated with readmission (*p* = 0.075).

**Table 1:** Sample characteristics^a^

Characteristic	Total sample N (%) or mean (SD)	Readmitted N (%) or mean (SD)		*p*-value
		Yes	No	
Total	477,964 (100%)	38,734 (8.1%)	439,230 (91.9%)	
*Sex*				< 0.001
Male	230,350 (48.2%)	19,111 (8.3%)	211,419 (91.7%)	
Female	247,613 (51.8%)	19,623 (7.9%)	227,990 (92.1%)	
*Race*				< 0.001
White	319,238 (66.8%)	26,279 (8.2%)	292,959 (91.8%)	
Asian	13,662 (2.9%)	1094 (8.0%)	12,568 (92.0%)	
Black	40,803 (8.5%)	3839 (9.4%)	36,964 (90.6%)	
Hispanic	26,065 (5.5%)	2517 (9.7%)	23,548 (90.3%)	
Other	2128 (0.4%)	197 (9.3%)	1931 (90.7%)	
Unknown	76,068 (15.9%)	4808 (6.3%)	71,260 (93.7%)	
Age	63.2 (13.8)	63.1 (14.9)	63.2 (13.7)	0.124
Body mass index	28.8 (7.4)	28.5 (8.0)	28.8 (7.4)	< 0.001
Comorbidity	313,756 (65.6%)	28,504 (9.1%)	285,252 (90.9%)	< 0.001
*Wound classification*				< 0.001
Clean	181,243 (37.9%)	6986 (3.9%)	174,257 (96.1%)	
Clean/Contaminated	215,729 (45.1%)	22,145 (10.3%)	193,584 (89.7%)	
Contaminated	40,684 (8.5%)	4871 (12.0%)	35,813 (88.0%)	
Dirty/Infected	40,308 (8.4%)	4732 (11.7%)	35,576 (88.3%)	
*Surgery*				< 0.001
Total hip replacement	175,221 (36.7%)	6526 (3.7%)	168,695 (96.3%)	
Coronary artery bypass grafting	8733 (1.8%)	727 (8.3%)	8006 (91.7%)	
Ivor lewis esophagectomy	3482 (0.7%)	399 (11.5%)	3083 (88.5%)	
Pancreaticoduodenectomy	21,108 (4.4%)	3763 (17.8%)	17,345 (82.2%)	
Distal pancreatectomy	9087 (1.9%)	1471 (16.2%)	7616 (83.8%)	
Pneumonectomy	4598 (1.0%)	385 (8.4%)	4213 (91.6%)	
Colectomy	255,735 (53.5%)	25,463 (10.0%)	230,272 (90.0%)	
*ASA Class*				< 0.001
Class 1	9943 (2.1%)	383 (3.9%)	9560 (96.1%)	
Class 2	192,466 (40.3%)	10,794 (5.6%)	181,672 (94.4%)	
Class 3	238,154 (49.8%)	22,969 (9.6%)	215,185 (90.4%)	
Class 4	35,887 (7.5%)	4459 (12.4%)	31,428 (87.6%)	
Class 5	1514 (0.3%)	129 (8.5%)	1385 (91.5%)	
Length of stay	5.4 (6.2)	7.4 (6.1)	5.3 (6.2)	< 0.001
Total hip replacement	2.2 (2.7)	3.3 (3.5)	2.2 (2.7)	< 0.001
Coronary artery bypass grafting	9.8 (6.1)	10.7 (6.0)	9.7 (6.1)	< 0.001
Ivor Lewis esophagectomy	10.7 (6.9)	9.8 (4.6)	10.8 (7.2)	0.948
Pancreaticoduodenectomy	10.3 (6.9)	9.7 (4.8)	10.5 (7.2)	0.173
Distal pancreatectomy	6.9 (5.3)	7.1 (4.3)	6.8 (5.4)	< 0.001
Pneumonectomy	7.1 (5.8)	7.4 (5.1)	7.0 (5.9)	< 0.001
Colectomy	6.9 (6.9)	7.9 (6.5)	6.8 (6.9)	< 0.001
*Discharge destination*				< 0.001
Home	420,737 (88.0%)	32,559 (7.7%)	388,178 (92.3%)	
Another care facility	50,656 (10.6%)	6037 (11.9%)	44,619 (88.1%)	
Expired	6049 (1.3%)	–	–	
Infectious complication	55,700 (11.7%)	16,352 (29.4%)	39,348 (70.6%)	< 0.001
30-Day mortality	8451 (1.8%)	724 (8.6%)	7727 (91.4%)	0.075

^a^SD = Standard Deviation

### Multivariate generalized linear mixed regression analysis of 30-day readmission

**Fig. 2:**
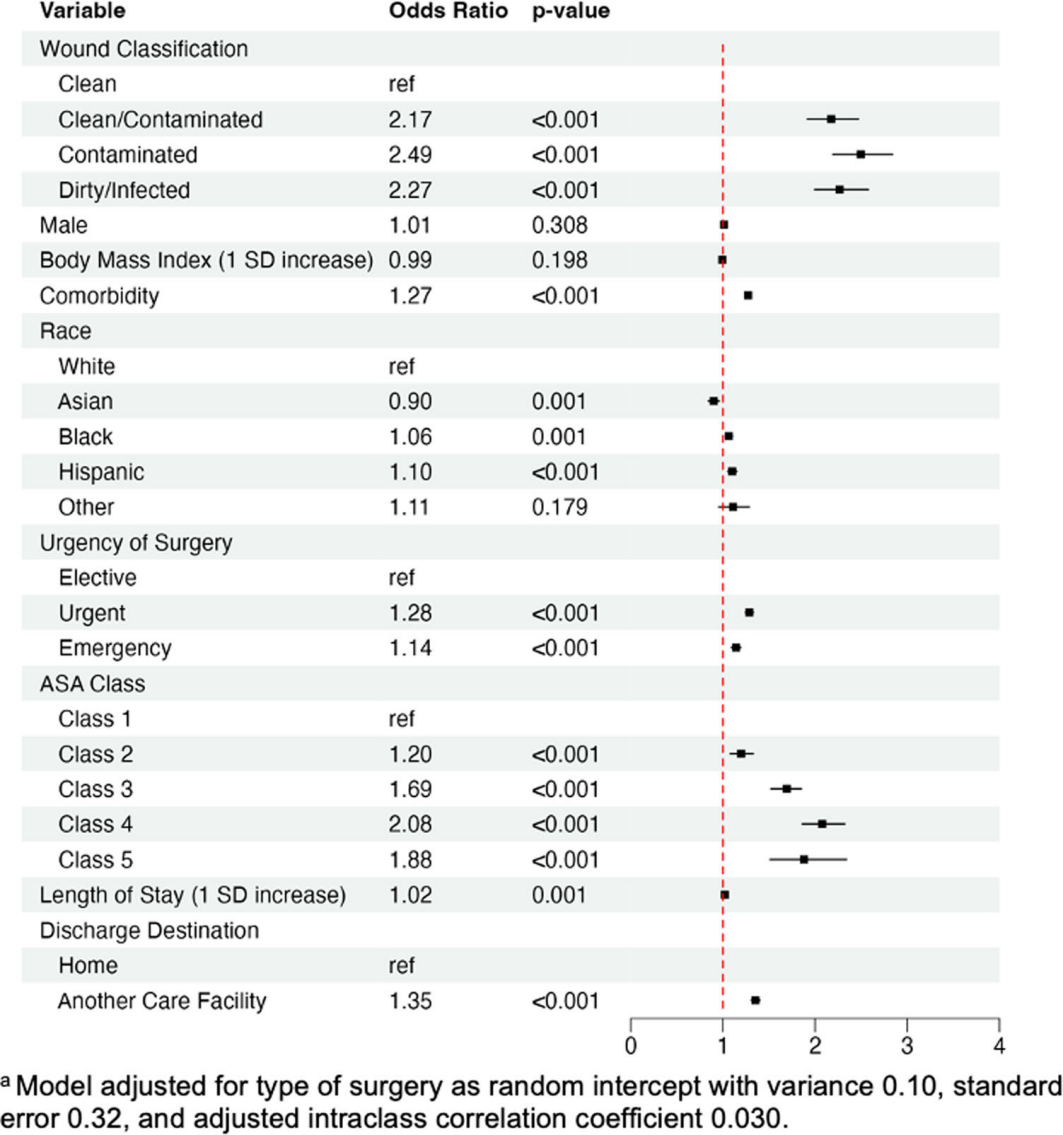
Multivariable model analysis of 30-Day readmission

The multivariate generalized linear mixed regression model of 30-day readmission on wound classification adjusted for sex, BMI, race, ASA class, presence of comorbidity, length of stay, urgency of surgery, and discharge destination as fixed effects and type of surgery as a random intercept (Fig. 2). Surgery type had an adjusted intraclass correlation coefficient of 0.030, indicating that it explains 3.0% of the total variance in 30-day readmission after adjusting for fixed effects. Of the covariates analyzed, wound class and ASA class had the strongest adjusted odds ratios, indicating elevated risk of readmission with “non-clean” wound class and higher ASA class. When compared to “clean,” the surgeries of “clean/contaminated,” “contaminated,” and “dirty/infected” wound classes were estimated to have 2.17, 2.49, and 2.27 times (respectively) greater risk for readmission (p < 0.001 for all). Other characteristics associated with significantly increased risk of readmission included increased BMI, presence of comorbidity, Hispanic or Black ethnicity, increased length of stay, urgent or emergency surgery, and discharge to another care facility (when compared to patients discharged to home).

### Most common reasons for readmission

**Fig. 3:**
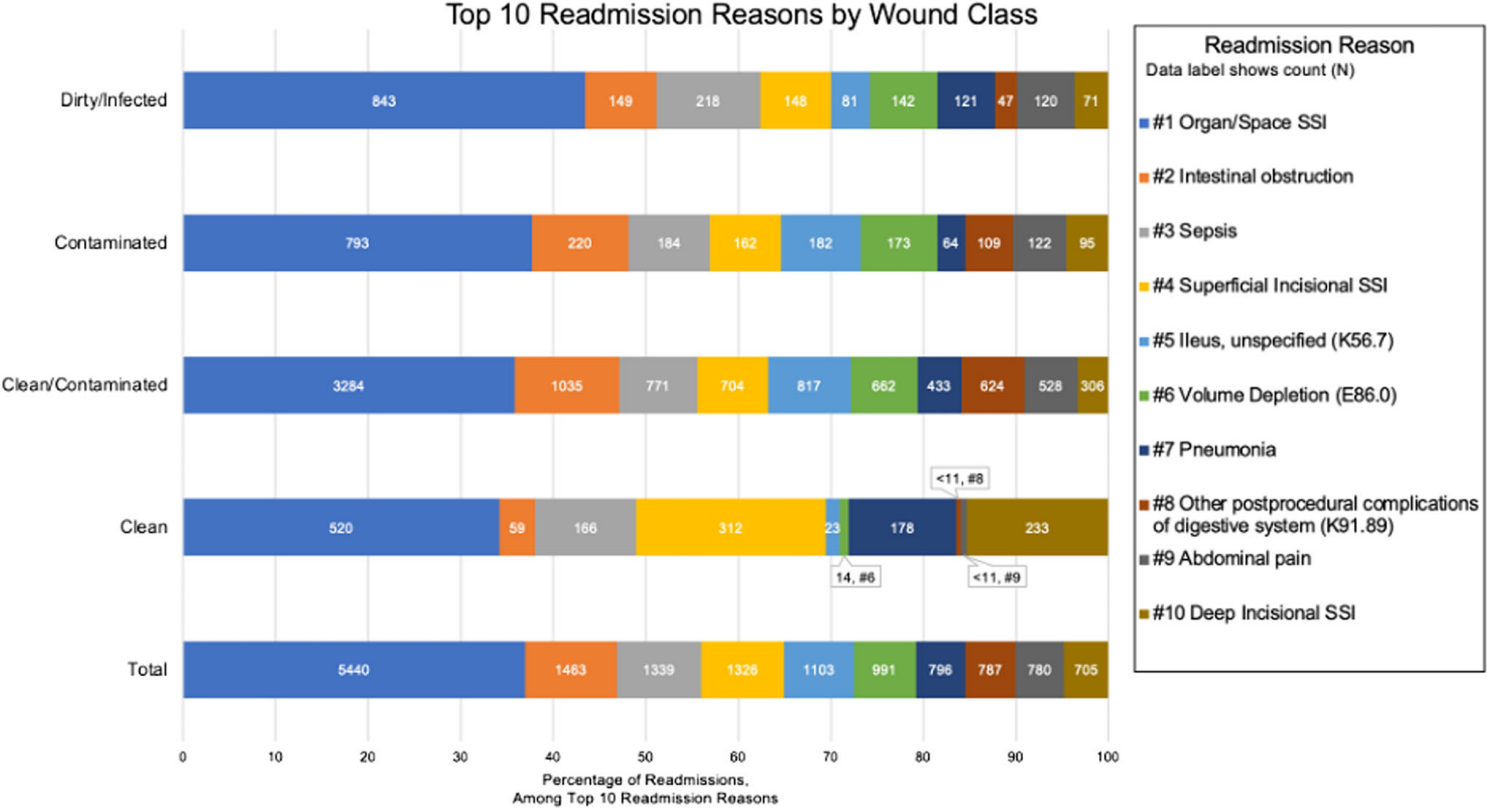
Top ten reasons for readmission with distributions by wound class

Among the 38,734 readmitted patients (8.1% of our total sample), 6,986 (18.0%) had undergone a “clean” surgery, 22,145 (57.2%) had “clean/contaminated” surgery, 4,871 (12.6%) had “contaminated” surgery, and 4,732 (12.2%) had “dirty/infected” surgery. The top ten reasons for readmission were explored. Among patients readmitted for one of the top ten readmission reasons in our sample, the proportion of each of the top ten reasons by wound class is displayed in Fig. 3. The most common reason for readmission was organ/space SSI, which appears to be more prevalent among “clean/contaminated,” “contaminated,” and “dirty” surgeries compared to “clean.” Other common reasons for readmission include intestinal obstruction, sepsis, superficial SSI, ileus, volume depletion, pneumonia, other postprocedural complications of digestive system, and deep SSI.

### Multivariate logistic regression analysis of postoperative complications

The associations between wound classification and organ/space SSI, deep SSI, superficial SSI, sepsis, or pneumonia in multivariate models were analyzed, as these outcomes were some of the most common reasons for readmission (Table 2). Compared to “clean” surgeries, “clean/contaminated”, “contaminated”, and “dirty/infected” wound classes were estimated to be 6.0, 9.3, and 18.0 times (respectively) as likely to experience organ/space SSI, the most common cause of readmission. In addition, dirtier wound classes, especially “contaminated” or “dirty/infected” were at significantly greater risk for sepsis, deep incisional SSI, and superficial SSI in multivariate models. Postoperative pneumonia was not significantly associated with wound class in the model.

**Table 2:** Adjusted odds ratio for postoperative complications by wound classification ^a,b^

Outcome	Wound classification			
	Clean	Clean/Contaminated	Contaminated	Dirty
	OR (95% CI)	OR (95% CI)	OR (95% CI)	OR (95% CI)
Organ/Space SSI	ref	6.0 (5.1, 7.1)	9.3 (7.8, 11.0)	18.0 (15.1, 21.2)
Deep incisional SSI	ref	5.2 (3.7, 7.1)	8.0 (5.7, 11.1)	7.8 (5.6, 10.7)
Superficial SSI	ref	1.3 (0.9, 1.7)	1.8 (1.3, 2.4)	1.8 (1.3, 2.4)
Sepsis	ref	3.6 (3.0, 4.4)	6.3 (5.1, 7.6)	22.8 (18.7, 27.6)
Pneumonia	ref	1.0 (0.6, 1.7)	1.3 (0.7, 2.2)	1.4 (0.8, 2.4)

^a^Multivariate model adjusted for wound classification, BMI, sex, race, ASA class, urgency of surgery, type of surgery, and presence of comorbidities

^b^OR = Odds Ratio; ref = reference group

## Discussion

This study of total hip replacement, CABGs, Ivor Lewis Esophagectomy, pneumonectomy, pancreaticoduodenectomy, distal pancreatectomy, and “dirty/infected” colectomy in the 2017–2020 ACS NSQIP database shows that dirtier wound classifications, when compared to “clean”, were at significantly higher risk for 30-day readmission. “Non-clean” wound classifications had one of the strongest adjusted odds ratios for 30-day readmission in the multivariate model which controlled for BMI, sex, race, ASA class, presence of comorbidity, urgency of surgery, length of stay, and discharge destination. Patients were most commonly readmitted for organ/space SSI, sepsis, pneumonia, and deep incisional SSI – complications related to infection. “Non-clean” wound class was also associated with significantly increased risk of these postoperative infectious complications in multivariate models.

Interestingly, “dirty/infected” wound class did not have a higher readmission risk than “clean/contaminated” or “contaminated” surgeries (when compared to “clean”). This may be a result of less physiologically complicated “dirty/infected” surgeries, as they were mostly represented by colectomies (Supplementary Table 2). Alternatively, it could be due to greater anticipation of postoperative infection when the surgical field has been grossly contaminated. Therefore, these patients may have had prolonged postoperative antibiotic which decreased readmission risk.

Wound classification had not previously been investigated as an important factor to consider for readmission risk among a broad group of surgeries. The wound classes were originally developed as a tool to estimate risk for surgical site infections and has been categorized as a "Must” document element at time of surgery by the CDC since 1985 [4]. In a study of 4,221 adrenalectomies, wound classification was not shown to be associated with readmission, however, the authors noted that adrenalectomy was typically a “clean” or “clean/contaminated” case, therefore a statistically significant difference between four wound class categories may not be detectable [14]. In another study examining 10,424 pediatric surgeries, dirtier wound class did not correlate with higher readmission rates nor with expected rates of SSI [15].

Readmission after surgery is an important outcome to investigate because CMS expanded its Hospital Readmission Reduction Program (HRRP) to include unplanned readmissions after total hip replacement and total knee replacements in 2015 and CABG in 2017 [1]. Hospitals with higher-than-expected readmission rates for certain index diagnoses or procedures are faced with decreased payments from Medicare [1]. This initiative has shown some success in reducing hospital readmissions and has saved Medicare hundreds of millions of dollars due to hospital penalties [1, 16].

The most significant finding of this study was the utility of wound class in prognosticating risk of 30-day readmissions following surgery. Patients undergoing surgeries with “non-clean” wound class may be at a statistically significant, greater than two-fold, risk of readmission when compared to “clean” surgeries (Fig. 2). If expansion of HRRP occurs to include more surgeries, CMS should recognize “non-clean” wound class as an important factor for readmission and postoperative infection, as they dictate permissible readmission rates. A range of readmission rates for a surgery, depending on its wound classification, could be implemented to avoid over-penalizing surgeries with inherently higher risks of readmission. Dissuading the approach to more complex and challenging patients may be the unintended consequence of not factoring in the wound classification. Another unintended consequence may be that surgeons would be incentivized to report their cases as contaminated. While surgeons strive to accurately report wound class to objectively determine a patient’s postoperative SSI risk, the process for correctly classifying wounds requires further improvement. Even with a structured operative debrief, a study found 41.5% of wound classes incorrectly documented [17].

Findings from Table 2 and Fig. 3 suggest that infectious complications could be driving readmissions. This is consistent with existing literature that reveals how SSI can lead to increased readmission rates, making it a potential target to improve quality measures [18, 19]. Dirtier wound class was significantly associated with surgical site infections and sepsis, which may explain why wound class was significantly prognostic of readmission in this study. In a case control study, prophylactic antibiotics was found to be protective of deep SSI during initial surgical admission [18]. Optimization of antibiotic use or source control among “non-clean” surgeries should be investigated further to examine its effect on readmission rate. Similar to our findings, one study showed that “contaminated” and “dirty/infected” abdominal wall reconstruction surgeries were independent predictors of surgical site infections [6]. Other studies determined that rates of superficial, deep, and/or organ/space SSI increased with dirtier wound classifications, consistent with how this study found dirtier wound classifications had elevated risk of SSI when adjusting for patient and clinical characteristics [7, 8, 11]. On the other hand, studies of pediatric surgeries did not show wound classification to accurately reflect the risk of SSI [5, 15]. Further investigation into the mechanism by which “non-clean” wound class elevates a patient’s risk for readmission is warranted. Our study could not examine the association of infectious complications on readmission in multivariate models as the timing of development of infection with readmission is not explicitly collected by ACS NSQIP.

This study has several limitations. The multivariate model of readmission does not take into account the hospital course (except for adjusting for length of stay and discharge destination) nor does it account for the indication for surgery (except for its urgency). These factors may confound the association between wound classification and readmission. Future studies should account for surgical indication and postoperative antibiotic use or surgical management. Secondly, inconsistencies in the reported wound classification in the ACS NSQIP database have been documented [20]. Although cases with miscoded wound class (based on definitions in Fig. 1) were excluded, there likely was some element of wound misclassification in this study. However, initiatives to document surgical site infection more accurately have emerged recently [17, 20]. Variability in readmission criteria could also bias the findings in this study, as hospitals, service lines and providers may have different thresholds for readmission. Lastly, hospital case mix could certainly influence readmission rates and future studies can investigate hospital-level data as confounding factors.

## Conclusion

Wound classification was strongly prognostic of 30-day readmission in multivariate models, suggesting that it may serve as a marker of readmissions after surgery. Increased risk of postoperative infectious complications associated with dirtier wound classifications may have contributed to this elevation in readmission risk. Since readmission following surgical procedures is commonly viewed as a quality-of-care measure and can result in penalties for hospitals, it is important to recognize that readmissions following a surgery cannot be viewed uniformly. Wound classification is one characteristic which highlights why a standardized readmission rate may not be an appropriate metric to evaluate the quality of surgical care.

## Supplementary information

 Below is the link to the electronic supplementary material.

Supplementary file1 (HTML 1 KB)

Supplementary file2 (XML 97 KB)
