# Supplementary material for: Centers for Disease Control (CDC) Wound Classification is Prognostic of 30-Day Readmission Following Surgery
Source: World J Surg. 2023 Jul 5;47(10):2392–400. doi: 10.1007/s00268-023-07093-3 (PMC10474202; doi:10.1007/s00268-023-07093-3)
Supplement: Supplementary file 1 — Supplementary file1 (HTML 1 KB) [file 268_2023_7093_MOESM1_ESM.html]

Manifest file for export wjs-23-02-0260-20230611155509


|  |  |
| --- | --- |
| File name: wjs-23-02-0260-20230611155509 | |
| Export Date: 11-Jun-2023 | |
| Output Format: XML (ScholarOne DTD) | |
| wjs-23-02-0260-20230611155509/doc/wjs-23-02-0260-File001.docx | Version 1.0 |
| wjs-23-02-0260-20230611155509/graphic/wjs-23-02-0260-File002.png | Version 1.0 |
| wjs-23-02-0260-20230611155509/graphic/wjs-23-02-0260-File003.png | Version 1.0 |
| wjs-23-02-0260-20230611155509/graphic/wjs-23-02-0260-File004.png | Version 1.0 |
| wjs-23-02-0260-20230611155509/graphic/wjs-23-02-0260-File005.png | Version 1.0 |
| wjs-23-02-0260-20230611155509/suppl\_data/wjs-23-02-0260-File006.docx | Version 1.0 |
| wjs-23-02-0260-20230611155509/suppl\_data/wjs-23-02-0260-File007.jpg | Version 1.0 |
| wjs-23-02-0260-20230611155509/pdf/wjs-23-02-0260.pdf |  |
| wjs-23-02-0260-20230611155509/wjs-23-02-0260-metadata.xml |  |
| wjs-23-02-0260-20230611155509/s1.dtd |  |
| manifest.html | This document |
